# Updated restraint dictionaries for carbohydrates in the pyranose form

**DOI:** 10.1107/S2059798322001103

**Published:** 2022-03-04

**Authors:** Mihaela Atanasova, Robert A. Nicholls, Robbie P. Joosten, Jon Agirre

**Affiliations:** a York Structural Biology Laboratory, Department of Chemistry, University of York, York YO10 5DD, United Kingdom; b Structural Studies, MRC Laboratory of Molecular Biology, Francis Crick Avenue, Cambridge CB2 0QH, United Kingdom; c Biochemistry Department, Netherlands Cancer Institute, Amsterdam, The Netherlands; d Oncode Institute, The Netherlands

**Keywords:** dictionaries, restraints, carbohydrates, pyranose, ring conformation

## Abstract

Restraint dictionaries are used during macromolecular structure refinement to encapsulate intramolecular connectivity and geometric information. These dictionaries allow previously determined ‘ideal’ values of features such as bond lengths, angles and torsions to be used as restraint targets. During refinement, restraints influence the model to adopt a conformation that agrees with prior observation. This is especially important when refining crystal structures of glycosylated proteins, as their resolutions tend to be worse than those of non­glycosylated proteins. Pyranosides, the overwhelming majority component in all forms of protein glycosylation, often display conformational errors in crystal structures. Whilst many of these flaws usually relate to model building, refinement issues may also have their root in suboptimal restraint dictionaries. In order to avoid subsequent misinterpretation and to improve the quality of all pyranose monosaccharide entries in the CCP4 Monomer Library, new dictionaries with improved ring torsion restraints, coordinates reflecting the lowest-energy ring pucker and updated geometry have been produced and evaluated. These new dictionaries are now part of the CCP4 Monomer Library and will be released with *CCP*4 version 8.0.

## Abstract 2

New restraint dictionaries for carbohydrates in the pyranose form have been produced using the latest methods as released in *CCP*4. The new restraints allow users to keep pyranoside models in their lowest energy conformation during refinement.

## 1. Introduction

Macromolecular refinement is a computational procedure that lies among the final steps in protein structure solution. Provided that a suitable strategy is selected, restrained refinement iteratively improves the agreement between a macromolecular model and experimental data. However, due to limited data resolution, prior chemical knowledge about the molecules involved is usually required in order to maintain refinement stability. In macromolecular crystallography, such prior knowledge is stored in dictionaries, typically in the form of Crystallographic Information Files (CIFs; Hall *et al.*, 1991 ▸; Brown & McMahon, 2002 ▸) or MOL(2) files (MDL Information Systems, San Leandro, California, USA). It is often the case that each molecular component (or residue, including carbohydrate monomers) is represented in a separate CIF. The restraint dictionary entries that are used by the software in the *CCP*4 suite (Winn *et al.*, 2011 ▸) are collected in the CCP4 Monomer Library (Vagin *et al.*, 2004 ▸). Such prior chemical knowledge usually consists of atom names, a description of stereochemical properties such as connectivity, bond lengths, angles, chirality, torsion angles and, if applicable, a list of groups of four or more atoms in planar co-arrangements.

This is especially important when modelling carbohydrates, which tend to be less well resolved due to flexibility, microheterogeneity, disorder (Joosten & Lütteke, 2017 ▸; Atanasova *et al.*, 2020 ▸) and relatively low data resolution (van Beusekom, Lütteke *et al.*, 2018 ▸). In addition, pyranosides, which are monosaccharides that form a six-membered saturated ring, can exhibit a range of different ring conformations. However, pyranosides are most frequently found in their lowest-energy conformation, a chair conformation (^4^ *C*_1_ for d-sugars and ^1^ *C*_4_ for l-sugars); this is particularly true for N-glycosylation (Agirre, Davies *et al.*, 2015 ▸). Chair conformations have minimal repulsions and strain due to the substituents being staggered rather than eclipsed, resulting in the dihedral angles between consecutive ring atoms being ±60°. Non-chair conformations and conformational transitions are costly in terms of energy and occur most frequently in enzymatic reactions (Davies *et al.*, 2012 ▸). Therefore, atomic models showing high-energy ring conformations need to be supported clearly by experimental data and shown in electron-density/potential maps (Agirre, 2017 ▸).

The CCP4 Monomer Library (CCP4-ML) was originally generated using the *LIBCHECK* software (Vagin *et al.*, 2004 ▸), which derived ideal values for saccharides from nucleic acid studies (Saenger, 1984 ▸). The CCP4-ML has seen recent expansion, and many component entries and linkages have now been replaced with *AceDRG* dictionaries (Nicholls, Joosten *et al.*, 2021 ▸). However, monosaccharides in pyranose form were set aside to be treated separately due to their particularities concerning ring conformation, which have recently been reviewed (Joosten *et al.*, 2021 ▸). Revision of these entries is thus overdue, not just as an effort to modernize geometric estimates, but also as a way of correcting issues that have been flagged up in the past (Agirre, 2017 ▸).

In general, restraint dictionary-generation programs use methods based either on data derived from small-molecule databases or on quantum-chemical calculations. Small-molecule-based dictionary-generation programs extract high-resolution geometric information from small-molecule databases in order to produce restraints for use in macromolecular crystallography. Examples of such small-molecule databases, which contain structural models that were derived using small-molecule X-ray crystallography, include the Cambridge Structural Database (CSD; paid access, 1 136 493 deposited structure models at the time of writing; Groom *et al.*, 2016 ▸) and the Crystallography Open Database (COD; free access, 473 816 deposited structure models at the time of writing; Gražulis *et al.*, 2012 ▸). Whilst both databases are curated, a recent study showed that *post hoc* validation checks need to be in place if it is intended to use the derived information to make reliable inferences regarding stereochemical geometries (Long *et al.*, 2017*a* ▸). *Mogul* (Bruno *et al.*, 2004 ▸) and *AceDRG* (Long *et al.*, 2017*b* ▸) utilize molecular-geometry information extracted from the CSD and the COD, respectively.

There are multiple contemporary restraint dictionary-generation programs, which use different combinations of databases and mining tools. *AceDRG* mines the COD, validating entries. It then compiles ‘*AceDRG* tables’ containing atom types, bond types and other information included in restraint dictionaries. These tables are distributed as part of the *CCP*4 software suite and are used during the restraint dictionary-generation procedure. *AceDRG* uses *RDKit* (http://www.rdkit.org) for internal molecular representation, from which it identifies atom types. Combined with the data from the aforementioned tables, this produces a restraint dictionary entry. Finally, *AceDRG* uses *RDKit* to generate multiple possible conformers, and chooses the one with the lowest free energy. *Grade* (Global Phasing), *Pyrogen* from *Coot* (Emsley *et al.*, 2010 ▸) and *eLBOW* (*phenix.elbow*; Moriarty *et al.*, 2009 ▸) can use *Mogul* (Bruno *et al.*, 2004 ▸) to mine the CSD. *Pyrogen* can also use the *CCP*4-distributed tables created by *AceDRG*. In addition to mining small-molecule databases, *eLBOW* can also use force fields to utilize quantum-chemical calculations. A default simple force field and the semi-empirical RM1/AM1 method (Dewar *et al.*, 1985 ▸; Rocha *et al.*, 2006 ▸) are both implemented internally and do not rely on external software or third-party resources. Full quantum-chemical calculations with a number of third-party quantum-chemistry packages are also available for use. These are useful when insufficient data about a particular chemistry are present in small-molecule databases.

Carbohydrate model-validation software such as *Privateer* (Agirre, Iglesias-Fernández *et al.*, 2015 ▸) uses a combination of established metrics (RSCC, average *B* factors) and carbo­hydrate-specific metrics (puckering coordinates, nomenclature checks) to identify problematic models. Further approaches to general ligand validation that are available in *CCP*4 have been discussed by Nicholls (2017 ▸). *Coot* includes ligand-validation features that allow the visual assessment of multiple metrics alongside associated percentile ranks relative to all X-ray structural models (Emsley, 2017 ▸). These include the RSCC (equation 1) of the ligand-omitted 2*mF*_o_ − *DF*_c_ map, the RSCC of the difference map, the number of atom pairs with unlikely contacts between them and the *Mogul Z*-worst score (comparing the value of a geometric parameter with data collected from the CSD). *Flatland Ligand Environment View* (Emsley, 2017 ▸) is a *Lidia* feature that shows the ligand in 2D for an alternative visualization of a ligand in its structural context, highlighting intermolecular interactions. Map sharpening can uncover missing features and is especially useful for flexible regions of the model. Finally, inspecting refined *B* factors may also provide a useful insight into model reliability, especially when comparing the *B* factors of proximal atoms (Masmaliyeva *et al.*, 2020 ▸).

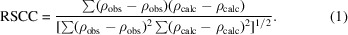

New dictionaries with improved ring torsion restraints, coordinates reflecting the lowest-energy ring pucker and updated geometry have been produced and evaluated using some of the metrics mentioned above. The new dictionaries, which are now part of the CCP4 Monomer Library, will be released with *CCP*4 version 8.0.

## 2. Materials and methods

### 2.1. Design guidelines

When using a restraint dictionary-generation program, the user needs to specify the molecular component for which a dictionary entry is to be generated. Typically, a pre-existing file that specifies chemical composition, connectivity and atomic nomenclature is used as input (for example using CIF, MOL or MOL2 format). In cases where such a file is unavailable, the chemistry can be specified using a Simplified Molecular-Input Line-Entry System (SMILES) string as input. SMILES is a linear notation that can represent 3D molecules as strings of characters (Weininger, 1988 ▸). Another option is to use a 2D sketcher, such as *Lidia* in *Coot* (Emsley, 2017 ▸) or *ChemDraw* (Perkin Elmer Informatics), or a 3D sketcher, such as *JLigand* (Lebedev *et al.*, 2012 ▸), to manually draw the molecule to either produce a SMILES string or otherwise a file that can be used as input.

Agirre (2017 ▸) analysed multiple restraint dictionary-generation programs by comparing the bond lengths and angles of the output for α-d-glucopyranose from a SMILES string with the ideal geometry described in the CCP4-ML. It was concluded that the dictionaries produced by *AceDRG*, *grade* and *eLBOW* using *Mogul* were roughly in agreement, meaning that they showed similar deviations from the targets proposed in the CCP4-ML. Recently, this observation was confirmed in a wider study, which showed that modern restraint dictionary-generation programs now show consistent results for carbohydrates in the pyranose form (Joosten *et al.*, 2021 ▸). Since *AceDRG* is the *CCP*4 program that is already being used to update dictionaries for other peptide, nucleotide and nonpolymeric chemical compounds in the CCP4-ML (Nicholls, Wojdyr *et al.*, 2021 ▸), as well as for chemical linkages between components, it was chosen to generate the restraint dictionary entries reported herein.

*AceDRG* allows different input options for the molecule to be generated. As mentioned above, a SMILES string is commonly used as input when no restraint dictionary entry is already available for a given molecular component, which is a common scenario during drug discovery. However, pyranose sugars follow the atomic nomenclature established by the Worldwide Protein Data Bank (wwPDB; Burley *et al.*, 2019 ▸) in its Chemical Component Dictionary (CCD; Westbrook *et al.*, 2015 ▸), which in turn now mirrors IUPAC nomenclature following the recent remediation of carbo­hydrate entries. In contrast, restraint dictionary entries produced from SMILES strings do not follow this convention (as atom nomenclature is not encoded in SMILES) and thus may end up causing issues during model building and refinement. For this reason, existing component definitions from the CCD were used as a starting point. These CIF files contain a description of the compound in terms of atom names, types and connectivity. Additionally, many component definitions contain idealized atomic coordinates from quantum-mechanics calculations. However, these coordinates do not provide sufficient information to construct restraints; any derived restraint target would lack an estimated standard deviation, which would be needed for relative weighting during refinement. Moreover, it has been found that restraints derived from QM-based calculations are at present inconsistent with those mined from high-quality small-molecule X-ray structures (Joosten *et al.*, 2021 ▸).

During the restraint dictionary-generation process for pyranose sugar entries, it is necessary to ensure that the anomeric configuration and the stereochemistry of the substituents are correct, and that the Cremer–Pople puckering coordinates that define the conformation of the pyranose ring in the conformer (Cremer & Pople, 1975 ▸) reflect a minimal energy ring pucker representative of the majority use case due to the rigid conformational preferences that saturated rings exhibit. As per the recommendations proposed by Joosten *et al.* (2021 ▸), restraint dictionary entries should present coordinates that are as close as possible to the most probable conformer. Torsion restraints, if present, should match these coordinates, allowing the refinement software to restrain the ring conformation to a minimal energy pucker at low resolution. *Privateer* (Agirre, Iglesias-Fernández *et al.*, 2015 ▸) was used to analyse the produced coordinates; this ensured that the stereochemistry, anomeric configuration and puckering parameters met expectations for each particular compound. As a secondary sanity check, all of the produced sugar monomers were visually inspected in *Coot* (Emsley *et al.*, 2010 ▸) to confirm that the stereochemistry of the substituents, the anomeric configuration and the conformation meet expectations. Furthermore, for those sugars where a protein crystal structural model derived using data extending to 1.5 Å resolution or better was available, the conformer presented in the new restraint dictionary entry was visually compared with the crystal structural model for further validation.

It is important to note that the atomic coordinates listed in the restraint dictionary are only used when initially placing the sugar into the model. Having reasonable starting coordinates, *i.e.* a low-energy conformer, is important in order to ensure a sensible starting point from which further model building and refinement can proceed. Any large deviation of the initial coordinates from the restraint targets causes imbalance in the refinement target function, causing slow and possibly sub­optimal refinement. Once the model is under refinement, these initial coordinates are discarded and it is the restraints themselves that continue to ensure a reasonable conformation. Consequently, it is of primary importance for the restraints to adequately reflect the allowable geometric landscape of the component. The inclusion of unimodal ring torsion restraints helps to ensure the maintenance of a low-energy ring conformation throughout the refinement procedure, except in cases where there is strong evidence to the contrary.

### 2.2. Protocol for generating new dictionaries

A set of pyranosides was obtained from the list of monosaccharides supported by *Privateer* (obtainable by running privateer -list on the command line); this set comprises the most frequently modelled pyranosides. CCD CIF files containing existing pyranoside definitions were downloaded from the PDB. These files were provided as input to *AceDRG* (version 231). Additionally, various *AceDRG* options were explored to avoid unexpected results such as distorted conformers. *AceDRG* samples many potential conformers; those with the lowest energy according to *RDKit* are optimized using the idealization mode of *REFMAC*5 (Murshudov *et al.*, 2011 ▸), and ultimately that with the lowest energy is selected (Long *et al.*, 2017*b* ▸). Specifying for a greater number of conformers to be sampled results in a noticeably increased computation time. The new restraint dictionary entry for each monosaccharide was output as a CIF file, along with a PDB file containing coordinates corresponding to a low-energy conformer. The PDB files for all entries were provided as input to *Privateer* for validation (Supplementary Table S1) and to *Coot* for further visual inspection. A compilation of all *Privateer* validation data is presented in Supplementary Table S1. Furthermore, in order to allow the use of predefined restraints for glycosidic linkages from the CCP4-ML, the component types were set to ‘pyranose’ for aldopyranoses and ‘ketopyranose’ for ketopyranoses (Nicholls, Wojdyr *et al.*, 2021 ▸). Adding the correct type is necessary as the glycosidic linkage restraints assume standard atom-naming conventions for the atoms involved, which are different for aldopyranoses and ketopyranoses.

**Figure 1:**
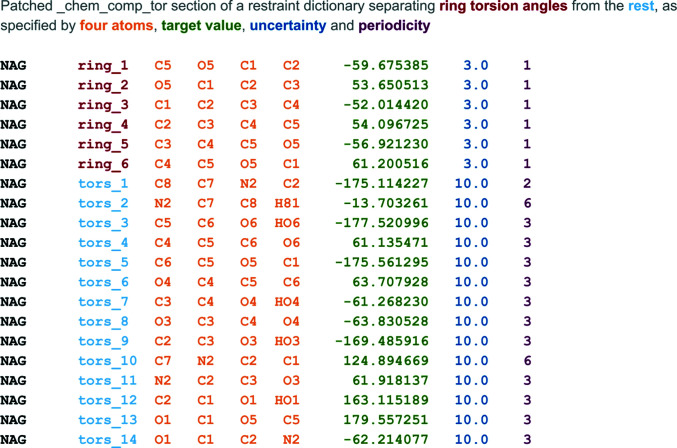
A view of the patched torsion section in a CIF restraint dictionary entry. This is an extract of the new CCP4 restraint dictionary entry for *N*-acetyl-β-d-glucosamine (GlcNAc), which is represented in the PDB as ‘NAG’. The new dictionaries distinguish ring torsion angles (prepended by ‘ring_’) from the rest (‘tors_’) so they can be activated separately to keep a low-energy ring pucker. Older CCP4 dictionaries had no separation between the ring torsions (unimodal) and the rest of the torsions (periodicity 2, 3 or 6), and had a uniform uncertainty of 20.0°.

The default torsion restraints generated by *AceDRG* do not exactly match the coordinates of a conformer, for example a generic 60° *versus* 53.65° as measured along O5–C1–C2–C3 for an energy-minimized conformer of *N*-acetyl β-d-glucosamine. While 60° may be appropriate for a carbon-only saturated ring such as cyclohexane, the presence of an endocyclic O atom in pyranosides means that not all bond lengths are the same, and therefore the torsion angles will reflect these differences. In order to address this potential shortcoming, *Privateer* has recently been extended to patch any restraint dictionary entry with torsion restraints that are measured from the Cartesian coordinates, writing separate names for ring torsions and other torsions so they can be used separately or together (Fig. 1 ▸). This functionality was used to patch the torsion restraints in the new dictionaries. The restraints called ‘ring_1’ to ‘ring_6’ (shown in bold) are the ring torsion restraints responsible for enforcing the minimum-energy ring conformation of the monosaccharide. Different sigmas for the ring torsions were tested (3.0°, 6.0° and 10.0°), selecting 3.0° as the value that yielded the fewest conformational outliers without having a detrimental impact on *R*_free_. Outliers at higher sigma levels were manually inspected and found to be unsupported by the electron density, meaning that they should have been corrected by the torsion restraints. All ring torsions were therefore set to 3.0°, with the rest of the restraints left at the *AceDRG* default value of 10.0°. For reference, the sigmas in the *LIBCHECK*-generated CCP4-ML dictionaries were all 20.0°; indeed, most restraints in the new dictionaries now show smaller sigmas than those in the *LIBCHECK*-generated CCP4-ML dictionaries.

### 2.3. Testing the new dictionaries

As has been described elsewhere (Agirre *et al.*, 2017 ▸), errors in carbohydrate models may be caused by incorrect model building: for example, if a monosaccharide is wrongly identified and ends up being distorted into the electron-density/potential map, or when the restraints used are insufficient to ensure reasonable geometry during refinement. Improved restraint dictionaries are expected to help to prevent issues with refinement. On the other hand, they will in no way avoid modelling mistakes. Such mistakes may be corrected either manually using available prior glyco-chemical knowledge (Bagdonas *et al.*, 2020 ▸) or automatically using specialized tools (van Beusekom, Lütteke *et al.*, 2018 ▸; van Beusekom *et al.*, 2019 ▸). Previous conformational analyses of PDB glycan data showed that the proportion of distorted pyranosides increased with worsening resolution, spiking significantly in the 1.8–2.0 Å region (Agirre, Davies *et al.*, 2015 ▸). Keeping in mind that the frequency of modelling mistakes is much higher at low resolution (Kleywegt & Jones, 1995 ▸), a decision was made to limit the test data set to include only entries with nominal resolutions better than 2.0 Å. Many pyranosides in our list are present in a very limited set of published structures and were not featured in the test data set. Therefore, a decision was made to choose representatives from the most frequently modelled pyranosides: NAG, MAN, BMA, GLC, BGC, BOG, FUL, GAL, GLA and SIA. A test data set was then assembled from the 100 PDB models with the highest numbers of the aforementioned pyranosides under the 2.0 Å resolution limit. The new dictionaries were then tested by refining the selected structural models with *REFMAC*5 (Murshudov *et al.*, 2011 ▸), using previously optimized refinement settings (restraint weights, *B*-factor models and solvent-mask parameters) extracted from the PDB-REDO databank (van Beusekom, Touw *et al.*, 2018 ▸). Three separate refinement protocols were devised: refinement with the current (referred to hereafter as ‘old’) dictionaries, refinement with the new dictionaries without torsion restraints and finally refinement with the new dictionaries with activated unimodal torsion restraints for pyranoside ring bonds. The output structural models and maps were then analysed using *Privateer* and *Coot*. The resultant data set was divided into two parts: sugars that are part of N/O-glycosylation and ligands. The sugars that are part of N/O-glycosylation were further filtered by excluding monomers marked as ‘wrong anomer’ (a mismatch between the anomeric form specified by the three-letter code and what is in the structure) and glycans that cannot be found in GlyConnect, one of the glycomics databases supported by *Privateer* (Bagdonas *et al.*, 2020 ▸).

## 3. Results

A set of 243 new carbohydrate dictionaries has been produced with updated torsion restraints that encourage refinement software to retain the minimal energy ring pucker. Fig. 1 ▸ shows an updated torsion section, taken from the new CCP4-ML entry for *N*-acetyl-β-d-glucosamine (GlcNAc; CCD component ID NAG). These new torsion restraints are especially important when the experimental data extend to only low resolution; enforcing torsion restraints from the dictionaries forces the sugar ring into the most likely conformation. As expected, introducing additional restraints, in this case the torsional kind, may occasionally lead to a lower real-space correlation coefficient (RSCC) between the model and map. This simply reflects the fact that the refinement software is no longer able to (inappropriately) improve the model-to-map correlation at the expense of stereochemical geometry, for example unfavourable bond lengths or angles or inverted ring conformations that would require a massive expenditure of energy.

The test data set was composed of 955 structural models containing 11 291 sugar residues; 5620 of these sugars were covalently bound to protein as part of N/O-glycosylated structures and 5671 were ligands. Obsolete CCD entries (for example all disaccharides, which following the PDB remediation of carbohydrate entries are now described as linked monosaccharides) were not included in the set.

*Privateer* was run on all structures in the test data set and the results were analysed. The sugars in the test data set were split into the categories ‘old dictionaries’, ‘new dictionaries’ or ‘new dictionaries and torsions’ based on the dictionaries that were used during their refinement, and labelled as ‘yes’, ‘no’ or ‘check’ according to the *Privateer* validation report. *Privateer* assigns a ‘yes’ diagnosis when the anomeric configuration, chirality, Cremer–Pople puckering parameters and ring conformation of a sugar are those expected for its lowest-energy conformer. The use of ring conformation as a validation metric for pyranosides is attractive because it cannot be targeted directly by a minimization of bond length and angle distortion; indeed, a boat conformation, which *Privateer* would show as an outlier, may have close-to-ideal bond lengths and angles. If *Privateer* detects any problems, the sugar is marked as ‘no’. However, if the only issue detected is in the ring conformation, *Privateer* instead marks the sugar as ‘check’, in which case the user should check whether the high-energy conformation is supported by the electron density. *Privateer* contains a database of puckering parameters calculated from a manually curated set of conformers obtained from the PDB CCD and compared against CSD, COD and high-resolution PDB structures. Ring conformation is a useful validation metric for pyranosides; however, it needs to be used in combination with other metrics, particularly density-based metrics, whenever unimodal restraints have been used due to bias towards one conformation.

**Figure 2:**
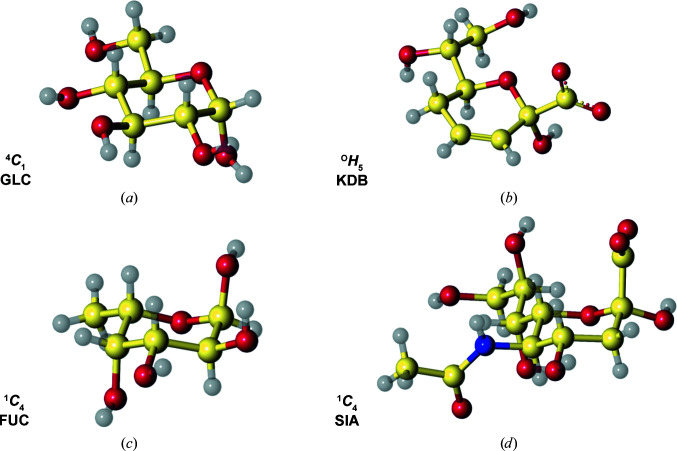
Carbohydrate restraint dictionary entries generated with *AceDRG*. (*a*) α-d-Glucose in the ^4^ *C*_1_ conformation, (*b*) 3,4,5-trideoxy-α-d-*erythro*-oct-3-en-2-ulopyranosonic acid in the ^O^ *H*_5_ conformation, (*c*) α-l-fucose in the ^1^ *C*_4_ conformation and (*d*) *N*-acetyl-α-neuraminic acid (sialic acid) in the ^1^ *C*_4_ conformation. This figure was produced with *CCP*4*mg* (McNicholas *et al.*, 2011 ▸).

The puckering parameters of the conformers stored in the dictionaries were also analysed using *Privateer*. All dictionaries show the expected puckering for their particular chemistry. For example, saturated rings show a chair conformation (^4^ *C*_1_ for d-pyranosides and ^1^ *C*_4_ for l-pyranosides) and pyrano­sides with a double bond in the ring, for example 3,4,5-trideoxy-α-d-*erythro*-oct-3-en-2-ulopyranosonic acid (CCD component ID KDB), show four coplanar atoms in the ring (see Fig. 2 ▸).

**Figure 3:**
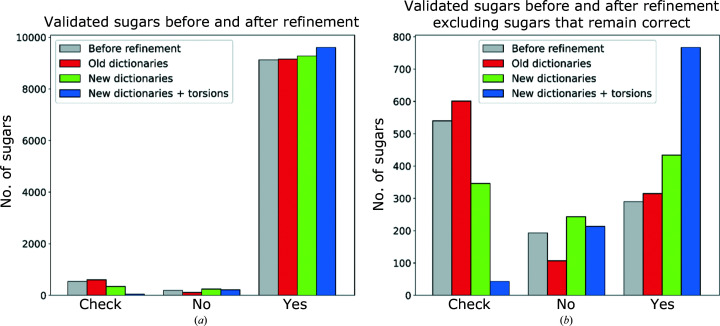
Numbers of sugars diagnosed by *Privateer* as ‘check’, ‘no’ and ‘yes’ before and after refinement. A set of structures from the PDB were refined with the CCP4-ML dictionaries, the new dictionaries generated by *AceDRG* and the new dictionaries with unimodal torsion restraints activated. From left to right, the coloured bars represent the number of sugars before refinement (grey) and the numbers of analysed sugars after refinement with the CCP4-ML dictionaries (red), after refinement with the new updated dictionaries (blue) and after refinement with the new dictionaries with activated unimodal torsion restraints (yellow). (*a*) shows all analysed pyranosides and (*b*) only includes pyranosides that were diagnosed as ‘check’ or ‘no’ for at least one protocol.

The number of pyranosides in each category was counted, the incorrect entries were excluded as described in Section 2 and the results are presented in Fig. 3 ▸. Fig. 3 ▸(*a*) includes all 9863 pyranosides from the test data set. In order to focus on the sugars where the new dictionaries have led to a change in behaviour, Fig. 3 ▸(*b*) only includes pyranosides validated as ‘check’ or ‘no’ in at least one of the test runs (1023 sugars). Sugars validated as correct in all three runs are deemed to be well supported by the experimental data and relatively easy to interpret. As expected, we registered a slight decrease in the RSCC, whereas both refinement protocols involving the new dictionaries, with and without torsion restraints activated, managed to reduce the gap between *R*_work_ and *R*_free_, indicating a reduction in overfitting (Supplementary Figs. S1–S3). In addition, there was a slight reduction in mean atomic *B* factors (Supplementary Fig. S4).

## 4. Discussion

Using *Privateer* for validation, we have sought to gain insight into how the quality of pyranoside models differs when using different restraint dictionaries during refinement. Three protocols were considered: using old dictionaries from the CCP4 Monomer Library, using new dictionaries generated using *AceDRG* and using new dictionaries with the addition of unimodal torsion restraints.

Fig. 3 ▸(*a*) shows that the great majority of pyranosides in the test set were correct before and after all three refinement protocols. This was expected, as previous research has shown that modelling errors increase greatly with decreasing resolution and particularly at resolutions lower than 2.0 Å (Atanasova *et al.*, 2020 ▸). Fig. 3 ▸(*a*) also shows that refinement with the old dictionaries produces very similar validation results to the original PDB models. This is somewhat surprising, as the original structures were produced using a variety of dictionaries and refinement software. Fig. 3 ▸(*b*) eliminates from the picture all of the structures that were correct in the original PDB models and continue to remain correct when using all three refinement protocols. The remaining cases indicate that the use of the old dictionaries reduces the number of residues validated as ‘no’ by *Privateer*, and moves the majority of these to the ‘check’ class (high-energy ring pucker but no other pathologies). In contrast, use of the new dictionaries (without activating torsion restraints) resulted in a slight decrease in the number of sugars diagnosed as ‘check’, indicating that the updated geometric estimates in the new dictionaries are sufficient to sway some models from a high-energy ring pucker into a chair conformation. This effect is greatly amplified when the new unimodal torsion restraints are activated; the number of pyranosides that change from showing one or more problems to being fully validated (a ‘yes’ diagnostic) almost doubles.

Some pyranosides remain incorrect after refinement with all three protocols. Upon closer inspection, most of these are cases where the electron density is difficult to interpret and often involve a modelling error. These cannot not be fixed by refinement alone, and in such cases additional intervention, for example interactive real-space refinement or running advanced rebuilding protocols such as those in *Rosetta* (Frenz *et al.*, 2019 ▸) or *Coot* (Emsley & Crispin, 2018 ▸), which are also automated in *PDB-REDO* (van Beusekom *et al.*, 2019 ▸), would be required in order to model the sugar correctly.

**Figure 4:**
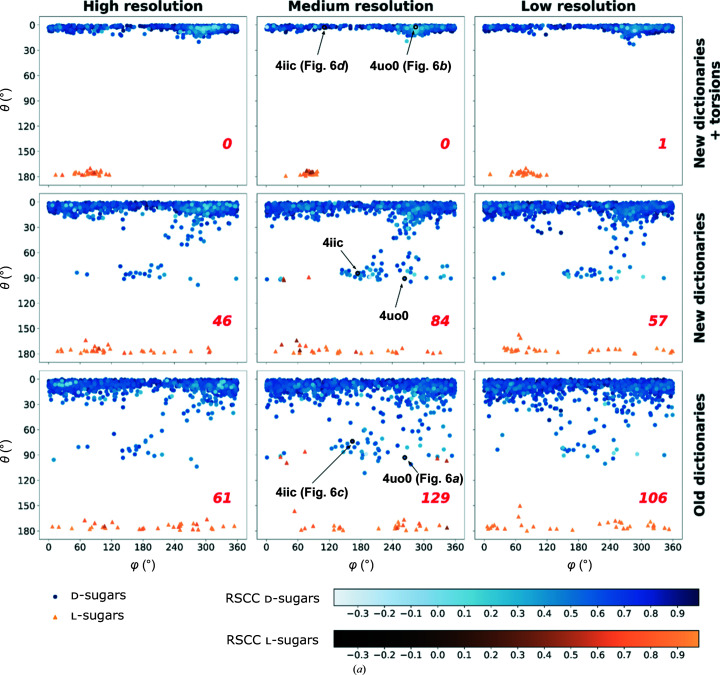

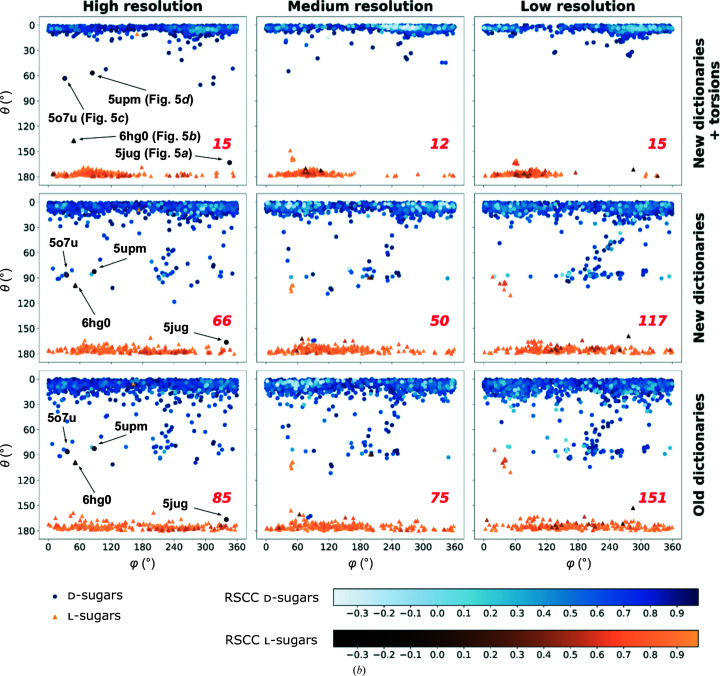
Refinement with the new dictionaries and unimodal torsion restraints leads to fewer unlikely carbohydrate conformations. (*a*) Sugars that are part of N/O-glycosylation; (*b*) other sugars. θ versus φ plot for d-sugars (blue circles) and l-sugars (yellow triangles); see Section 4 for a description of θ and the Cremer–Pople parameters. d-Sugars usually adopt the ^4^ *C*_1_ conformation with θ ≃ 0°; l-sugars normally adopt the ^1^ *C*_4_ conformation with θ ≃ 180°. Use of the new unimodal torsion restraints (top) shows fewer deviations from these values. The PDB codes corresponding to entries discussed in Figs. 5 ▸ and 6 ▸ are labelled. The number of sugars in high-energy conformations (according to *Privateer*) is shown in the bottom right corner of each plot. Resolution ranges contain equal numbers of sugars (1668 each). High resolution is 0.9–1.8 Å, medium resolution is 1.8–1.9 Å and low resolution is 1.9–2 Å.

In addition to diagnosing each pyranoside as ‘yes’, ‘no’ or ‘check’, *Privateer* also calculates the RSCC between the sugar-omitted ‘observed’ (2m*F*_o_ − *DF*_c_) electron-density map (ρ_obs_) and that calculated from the model (ρ_calc_) in the vicinity of the sugar (equation 1). Fig. 4 ▸ shows the change in the RSCC after refinement. The general trend is that the RSCC remains high overall. The new unimodal torsion restraints lead to an increase in the number of sugars validated as ‘yes’ and a decrease in the number of sugars diagnosed as ‘no’ or ‘check’. The average RSCC after refinement with the old dictionaries was 0.793, whereas with the new dictionaries it decreased to 0.791 and finally with the new dictionaries and unimodal torsion restraints it decreased further to 0.789 (Supplementary Figs. S2 and S3). As already discussed, restraining a sugar to the most likely conformation could lead to a small decrease in the RSCC. This is due to the sugar being encouraged to adopt a sensible conformation, rather than being allowed to sink into the electron density, however faint or incomplete, at the expense of unphysical geometric distortions. Such an avoidance of overfitting is generally the appropriate course of action (in the absence of clear evidence to the contrary). Indeed, a modest reduction of the RSCC should be seen as an acceptable trade-off when the electron-density map does not unambiguously demonstrate evidence for a high-energy conformation. Consistently, we also found a small but significant increase in Δ*R*_work_, while Δ*R*_free_ remained essentially the same with both refinement protocols involving the new dictionaries. This reduction in the gap between *R*_work_ and *R*_free_ (Supplementary Fig. S1) provides further evidence that using the new dictionaries can help prevent overfitting.

**Figure 5:**
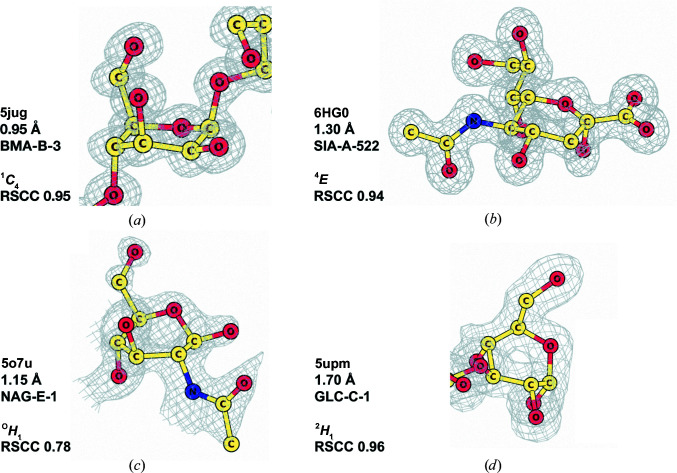
Sugars in unusual conformations after refinement with the new dictionaries with unimodal torsion restraints. (*a*) BMA-B-3 from PDB entry 5jug (Jin *et al.*, 2016 ▸); (*b*) SIA-A-522 from PDB entry 6hg0 (M. T. Salinger, J. R. Hobbs, J. W. Murray, W. G. Laver, P. Kuhn & E. F. Garman, unpublished work); (*c*) NAG-E-1 from PDB entry 5o7u (Tobola *et al.*, 2018 ▸); (*d*) GLC-C-1 from PDB entry 5upm (Pluvinage *et al.*, 2017 ▸). These sugars appear as outliers in Fig. 4 ▸(*b*). They remain in high-energy conformations after refinement, but have a high RSCC. This figure was produced with *CCP*4*mg* (McNicholas *et al.*, 2011 ▸). Maps are displayed at the 1σ contour level with a sampling rate of 0.5.

The θ angle of the Cremer–Pople parameters for pyranose rings (Cremer & Pople, 1975 ▸) is a useful tool in conformational analysis, as it helps to monitor the transition from chairs (θ ≃ 0° for ^4^ *C*_1_ chairs, θ ≃ 180° for ^1^ *C*_4_ chairs) into envelopes and half-chairs (θ ≃ 45° and θ ≃ 135°) and then into boats and skew-boats (θ ≃ 90°). As these transitions involve eclipsing of substituents and thus energy penalties, θ may be seen as a simple summary of the deviation of the geometric parameters of a sugar from ideal values. Examining the θ angle distribution provides further evidence to support the assertion that the unimodal torsion restraints decrease the number of un­likely conformations. Figs. 4 ▸(*a*) and 4 ▸(*b*) present a conformational analysis of all pyranosides in the test data set, binned into three resolution ranges (left, 0.9–1.8 Å; middle, 1.8– 1.9 Å; right, 1.9–2.0 Å). The three bins were chosen to contain the same number of pyranosides. As seen in both panels, the number of sugars with unusual θ angle values decreases significantly when the ring is restrained using the new uni­modal torsion restraints. Even the new dictionaries without torsions activated seemed to have a beneficial impact on ring conformation. Interestingly, the ligand pyranosides that remain in unusual conformations (Fig. 4 ▸ *b*) generally exhibit a high RSCC. A closer inspection of these outliers (Fig. 5 ▸) revealed that the conformations of these pyranosides are retained due to being supported by the data, as shown by strong and featureful electron-density maps. The relative weighting between the data and geometric components in *REFMAC*5 makes this possible, allowing torsion restraints to be down-weighted in favour of strong observations (Murshudov *et al.*, 2011 ▸). High-energy conformations such as these are usually adopted by ligands that are bound, or trapped, covalently linked in the middle of a reaction within an enzyme (Davies *et al.*, 2012 ▸).

**Figure 6:**
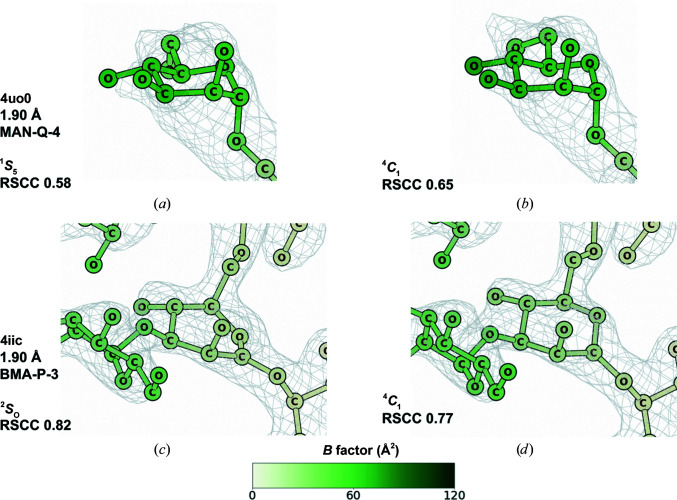
Change in conformation and real-space correlation coefficient (RSCC) after refinement. (*a*) Sugar in a ^1^ *S*_5_ conformation after refinement with its old CCP4-ML restraint dictionary entry (Fig. 4 ▸ *a*, bottom middle panel). (*b*) The conformation of the sugar has been changed to the minimal energy conformation after refinement with the updated restraint dictionary entry and unimodal torsion restraints and the RSCC has increased (Fig. 4 ▸ *a*, top middle panel). The sugar in (*a*) and (*b*) is MAN-Q-4 from PDB entry 4uo0 (Devi *et al.*, 2015 ▸) at 1.90 Å resolution, mean *B* value 34 Å^2^. (*c*) Sugar in a ^2^ *S*_O_ conformation after refinement with its old CCP4-ML restraint dictionary entry (Fig. 4 ▸ *a*, bottom middle panel). (*d*) The minimal energy conformation of the sugar after refinement with the new restraint dictionary entry and unimodal torsion restraints; the RSCC has decreased (Fig. 4 ▸ *a*, top middle panel). The sugar in (*c*) and (*d*) is BMA-P-3 from PDB entry 4iic (Suzuki *et al.*, 2013 ▸) at 1.90 Å resolution, mean *B* value 18 Å^2^. This figure was produced with *CCP*4*mg* (McNicholas *et al.*, 2011 ▸). Maps are displayed at a 1σ contour level with a sampling rate of 0.5.

Fig. 6 ▸ demonstrates the change in the RSCC when restraining pyranosides to their most likely ring conformations using the new dictionaries with unimodal torsion restraints. A pyranoside refined with the old CCP4-ML dictionaries is shown in Fig. 6 ▸(*a*), adopting an unlikely high-energy conformation (^1^ *S*_5_) with an RSCC of 0.58. When the conformation is moved to the more probable ^4^ *C*_1_ after refinement with the new dictionaries and unimodal torsion restraints (Fig. 6 ▸ *b*), the RSCC increases to 0.65, representing better agreement between the sugar and the electron-density map. The sugar shown in Fig. 6 ▸(*c*) is also in a high-energy conformation (^2^ *S*_0_) after refinement with the old dictionaries, which is corrected to ^4^ *C*_1_ after refinement with the new dictionaries and torsion restraints. However, in this case the RSCC decreases from 0.82 to 0.77. This once again demonstrates how restraining a sugar to the most likely conformation can have either an incremental or decremental effect on the RSCC, and that the RSCC is not always a helpful metric for assessing local model reliability. Finally, we should like to emphasize that while unimodal torsion restraints seem like a good tool for the refinement of pyranosides in general, they may mask other problems that can be detected by *Privateer* when they are not in use. Indeed, an unexpected high-energy ring pucker is considered to be a good indication of other modelling problems (Agirre, 2017 ▸).

## 5. Conclusion

As part of a recent overhaul of the CCP4 Monomer Library, in which existing dictionaries were replaced with those generated by *AceDRG*, we have augmented the dictionaries for pyranose entries by patching them with unimodal torsion restraints generated by *Privateer*. This development has the potential to dramatically reduce the number of conformational anomalies in refined structures. Users still have to be mindful of the need to activate torsion restraints in their respective refinement programs should they want to use them: they are currently deactivated by default in *CCP*4 software, although they may or may not be used automatically in other suites. To the best of our knowledge, this is the first time that torsional sets for pyranoses have been tested extensively in this manner and the results should give confidence that the torsion restraints in these dictionaries can lead to a chemically sensible result in the absence of serious modelling mistakes.

## 6. Open research data: availability and reproducibility

Our dictionaries will be released as part of the *CCP*4 suite with the release of version 8.0. The latest pre-release version of the CCP4-ML can be accessed by anonymous checkout (using the command bzr checkout https://ccp4serv6.rc-harwell.ac.uk/anonscm/bzr/monomers/trunk mon_lib). In addition to the new carbo­hydrate dictionaries, the previous CCP4 carbohydrate dictionaries (referred to in the text as ‘old’) at the time of publication and the results from all of the refinements can be downloaded from https://zenodo.org/record/5764924.

## Supplementary Material

Supplementary Figures and Table. DOI: 10.1107/S2059798322001103/rr5213sup1.pdf
