# Supplementary material for: Updated restraint dictionaries for carbohydrates in the pyranose form
Source: Acta Crystallogr D Struct Biol. 2022 Mar 4;78(Pt 4):455–65. doi: 10.1107/S2059798322001103 (PMC8972801; doi:10.1107/S2059798322001103)
Supplement: Supplementary file 1 [file d-78-00455-sup1.pdf]

STRUCTURAL
BIOLOGY

Volume 78 (2022)

Supporting information for article:

Updated restraint dictionaries for carbohydrates in the pyranose form

Mihaela Atanasova, Robert A. Nicholls, Robbie P. Joosten and Jon Agirre

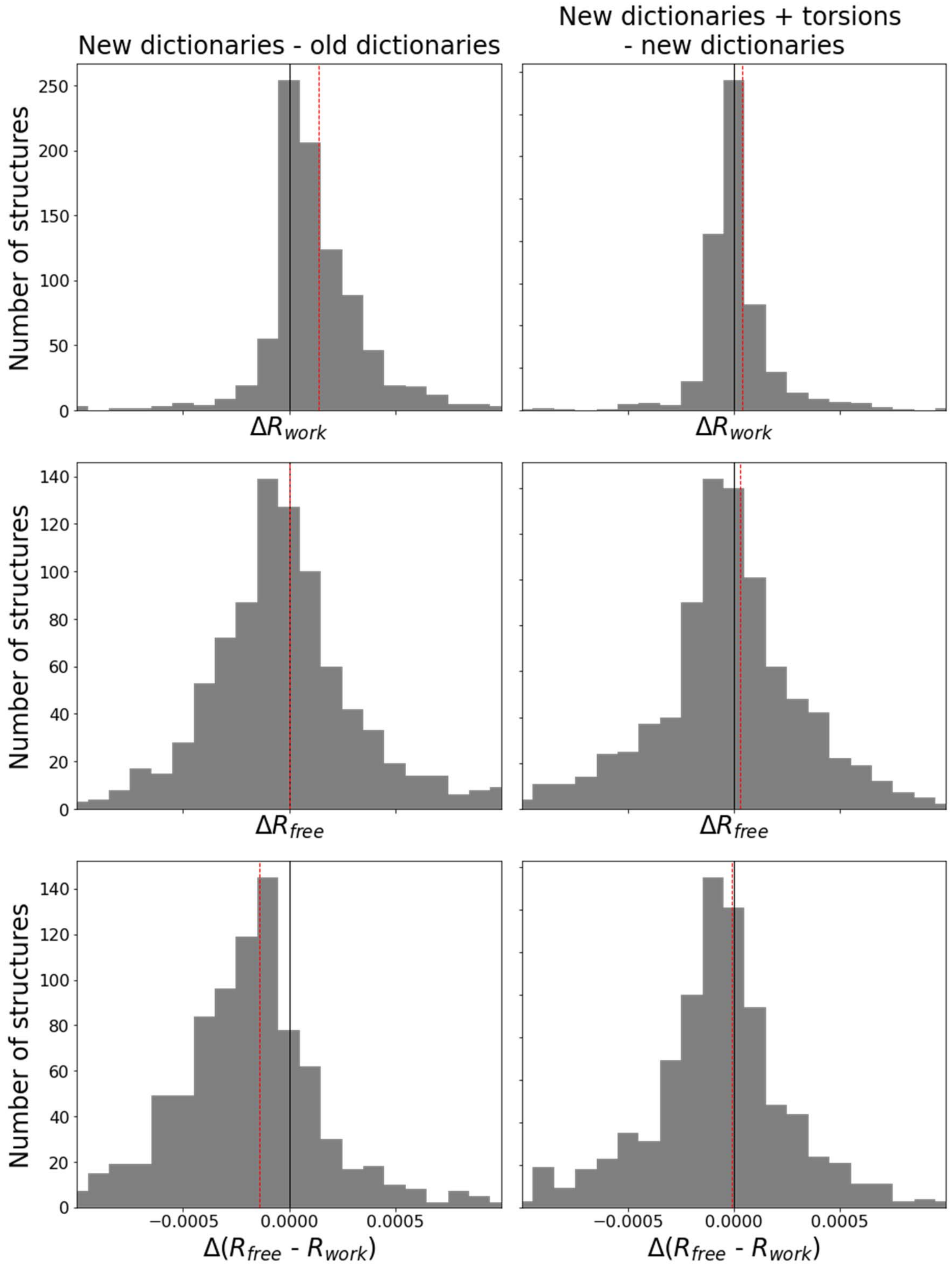

Figure S1 Distribution of changes in R_{work}, R_{free}, and the R-factor gap R_{work}-R_{free}. Black lines indicate the origin, and red dashed lines the median. The horizontal axis is truncated to the region of interest. Using the new dictionaries increases R_{work} while R_{free} is unchanged. As a result the R-factor gap is reduced indicating less overfitting in refinement. This effect is further strengthened, if to a lesser degree, when also including additional unimodal torsion restraints.

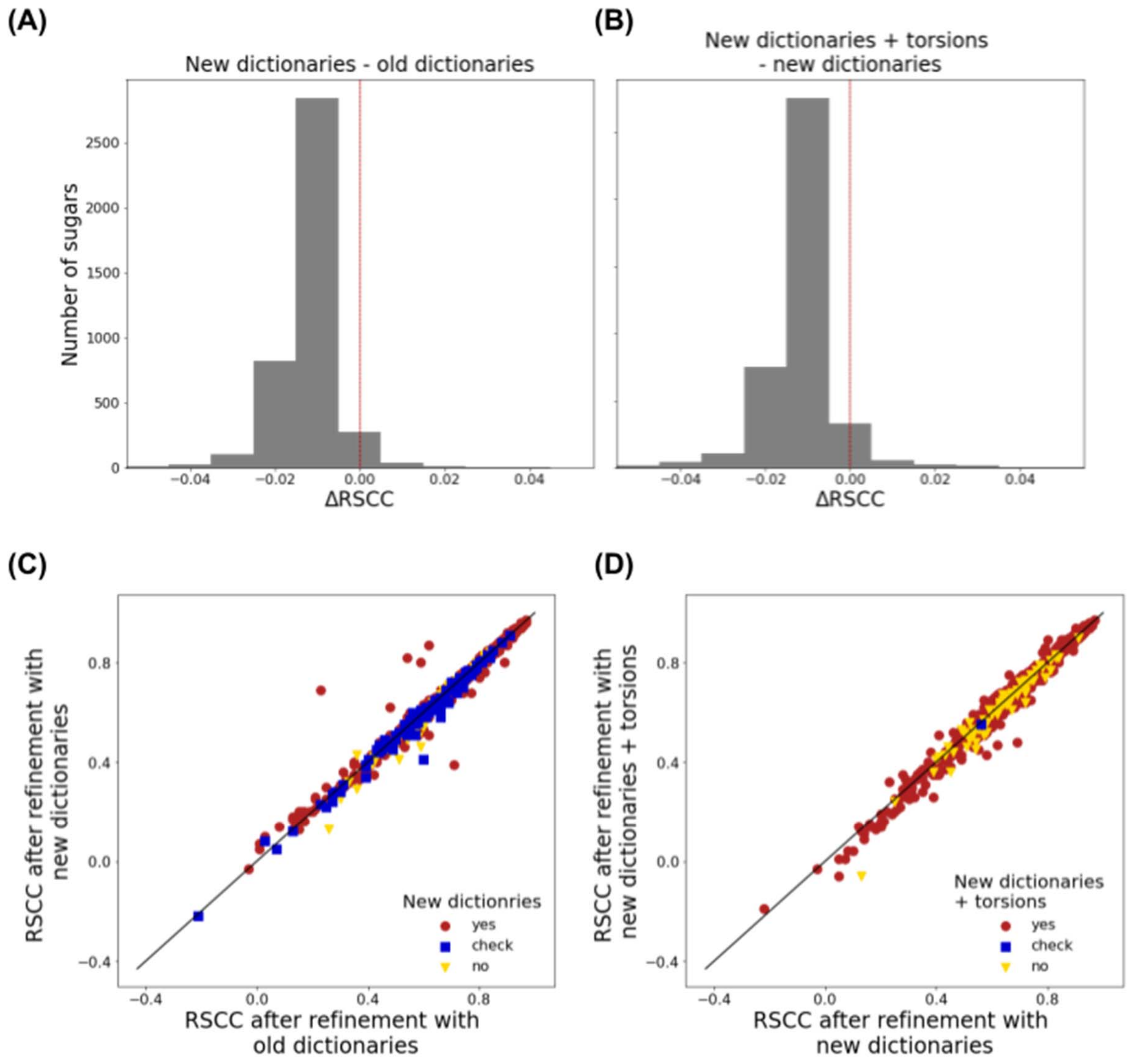

Figure S2 . There is a slight decrease in RSCC for sugars involved in N/O glycosylation after refinement with the new dictionaries, regardless of the use of unimodal torsion restraints. The sugars are marked as “yes”, “no”, and “check” based on their validation after refinement with the protocol on the vertical axis. The horizontal axis is truncated to the region of interest. In (A) and (B) black lines indicate the origin, and red dashed lines the median.

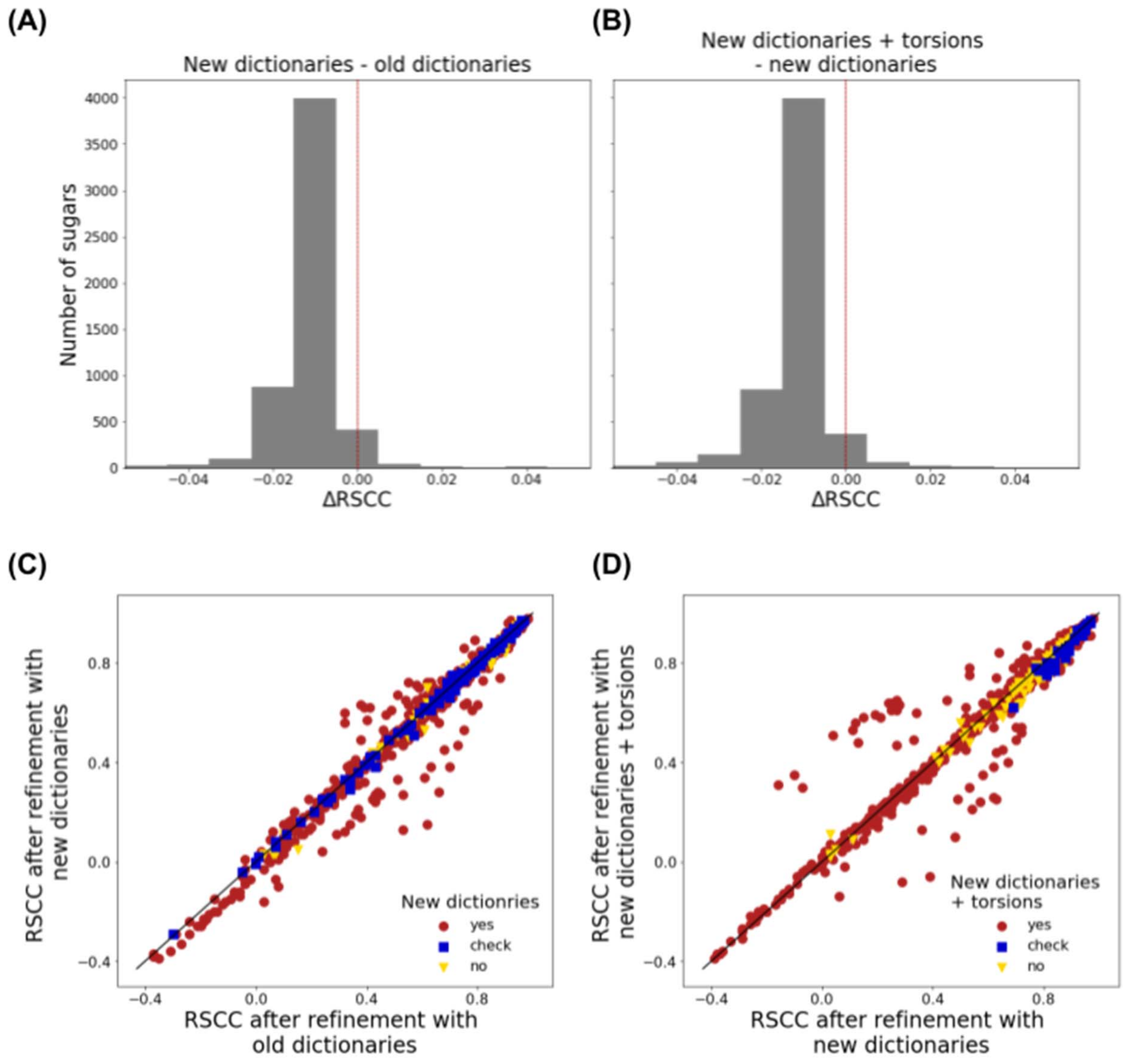

Figure S3 There is a slight decrease in RSCC for ligand sugars after refinement with the new dictionaries, regardless of the use of unimodal torsion restraints. We observe a bias in the distribution of outliers: a few isolated cases appear to achieve higher RSCC with the old dictionaries (C), while the same is true for the new dictionaries with torsions versus those without (D). These outliers are cases similar to that discussed in Figure 5. The sugars are marked as "yes", "no", and "check" based on their validation after refinement with the protocol on the vertical axis. The horizontal axis is truncated to the region of interest. In (A) and (B) black lines indicate the origin, and red dashed lines the median.

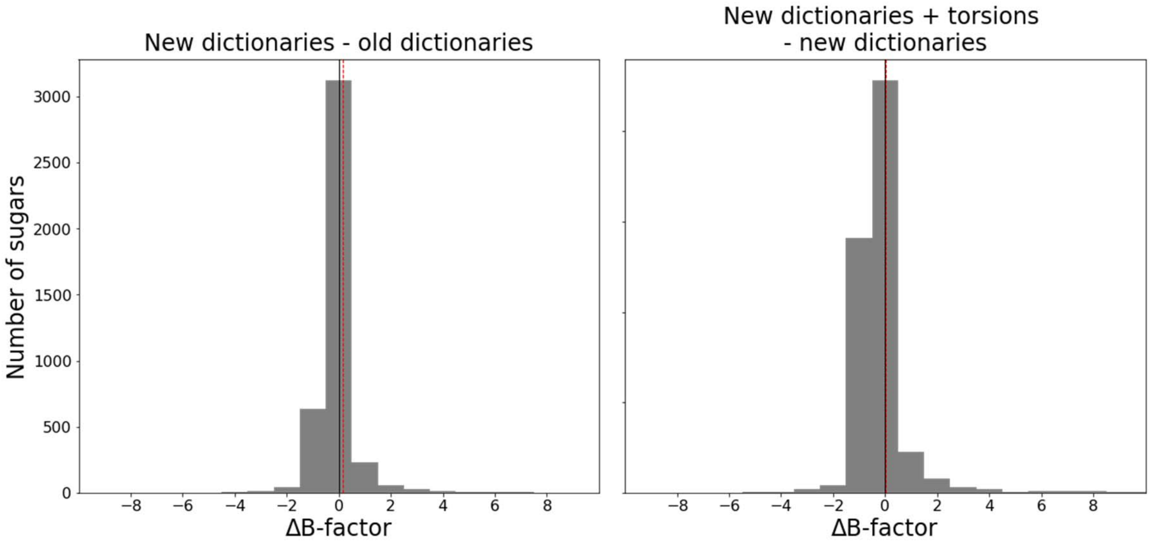

(A)

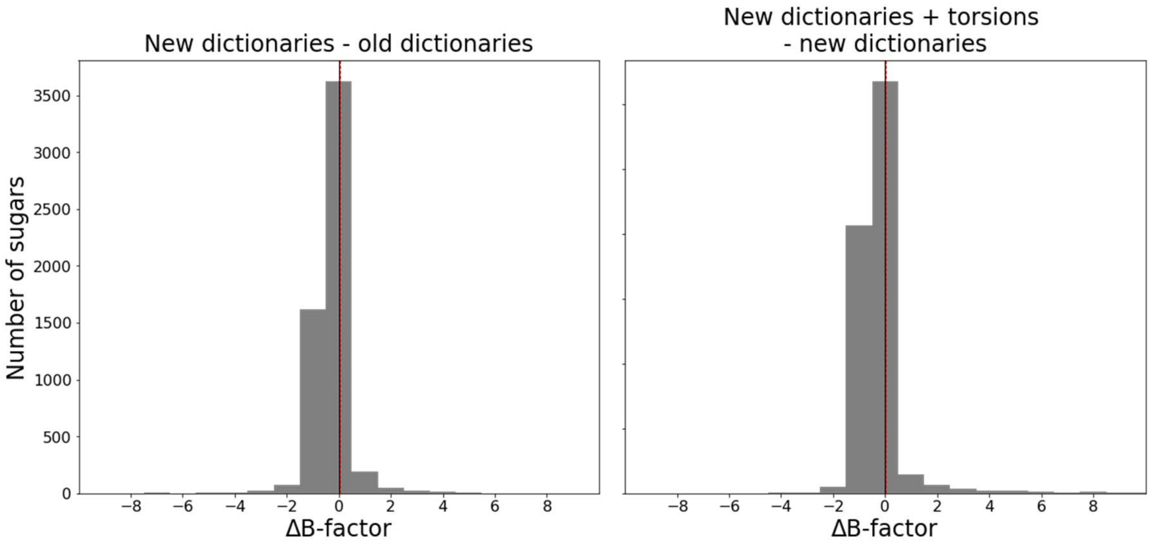

(B)

Figure S4 Distribution of changes in B-factor. The horizontal axis is truncated to the region of interest. Black lines indicate the origin, and red dashed lines the median. (A) Sugars that are part of N/O-glycosylation; (B) Ligands. There is a slight decrease in B-factor after refinement with the new dictionaries, and a further decrease when unimodal torsion restraints are used.

Table S1 Validation results for all the produced conformers as calculated by Privateer. Column legend: 'CCD' is the three-letter code assigned by the CCD; the Cremer-Pople parameters (Cremer & Pople, 1961), named 'Q', 'Phi' and 'Theta' describe pyranose ring conformation (denoted as 'Cnf' here), with 'Phi' and 'Theta' describing what atoms move away from the average ring plane, and 'Q' (termed 'total puckering amplitude' by Cremer & Pople, measured in Å^2) dictating by how much; 'Detected type' describes the monosaccharide in terms of anomeric form, absolute stereochemistry, position of the carbonyl group (aldose or ketose) and ring shape (all pyranose in this study); 'Ok?' presents the result of Privateer's tri-state validation diagnosis, as introduced in the main text; Lastly, 'name' is the full IUPAC name of the monosaccharide.

CCD	Q	Phi	Theta	Detected type	Cnf	Ok?	Name
145	0.574	344.33	3.93	beta-D-aldopyranose	4c1	yes	2-nitrophenyl beta-D-galactopyranoside
147	0.574	351.33	3.59	beta-D-aldopyranose	4c1	yes	4-nitrophenyl beta-D-galactopyranoside
16G	0.552	223.59	3.31	alpha-D-aldopyranose	4c1	yes	2-acetamido-2-deoxy-6-O-phosphono-alpha-D-glucopyranose
18D	0.542	53.26	175.51	alpha-L-ketopyranose	1c4	yes	3,5-dideoxy-5-(propanoylamino)-D-glycero-alpha-D-galacto-non-2-ulopyranosonic acid
1GL	0.541	259.05	4.43	alpha-D-aldopyranose	4c1	yes	2,6-dideoxy-4-O-methyl-alpha-D-galactopyranose
1GN	0.562	293.42	5.96	beta-D-aldopyranose	4c1	yes	2-amino-2-deoxy-beta-D-galactopyranose
289	0.549	271.25	5.57	beta-D-aldopyranose	4c1	yes	D-glycero-alpha-D-manno-heptopyranose
291	0.550	233.39	2.37	alpha-D-aldopyranose	4c1	yes	prop-2-en-1-yl 7-O-carbamoyl-L-glycero-alpha-D-manno-heptopyranoside
293	0.544	266.78	4.27	alpha-D-aldopyranose	4c1	yes	2-deoxy-beta-L-galacto-heptopyranose
2DG	0.547	268.84	5.72	alpha-D-aldopyranose	4c1	yes	2-deoxy-alpha-D-galactopyranose
2FG	0.562	290.35	4.65	beta-D-aldopyranose	4c1	yes	2-deoxy-2-fluoro-beta-D-galactopyranose
2GS	0.559	261.69	3.69	alpha-D-aldopyranose	4c1	yes	2-O-methyl-alpha-D-galactopyranose
3FM	0.538	333.45	1.10	alpha-D-aldopyranose	4c1	yes	3-O-carbamoyl-alpha-D-mannopyranose
3HD	0.574	328.99	2.30	beta-D-aldopyranose	4c1	yes	1,5-anhydro-3-O-methyl-D-mannitol

3MG	0.557	319.74	3.81	beta-D-aldopyranose	4c1	yes	3-O-methyl-beta-D-glucopyranose
42D	0.545	46.01	175.50	alpha-L-ketopyranose	1c4	yes	3,5-dideoxy-5-[(methoxycarbonyl)amino]-D-glycero-alpha-D-galacto-non-2-ulopyranosonic acid
445	0.562	10.77	4.37	beta-D-aldopyranose	4c1	yes	N-[oxo(phenylamino)acetyl]-beta-D-glucopyranosylamine
46M	0.567	295.18	5.81	beta-D-aldopyranose	4c1	yes	(4AR,6R,7S,8R,8AS)-hexahydro-6,7,8-trihydroxy-2-methylpyrano[3,2-D][1,3]dioxine-2-carboxylic acid
475	0.565	6.97	4.10	beta-D-aldopyranose	4c1	yes	N-[oxo(pyridin-2-ylamino)acetyl]-beta-D-glucopyranosylamine
49A	0.441	81.24	128.26	beta-L-ketopyranose	5h4	yes	4,9-amino-2,4-deoxy-2,3-dehydro-n-acetyl-neuraminic acid
4AM	0.443	82.25	128.10	beta-L-ketopyranose	5h4	yes	4-amino-2-deoxy-2,3-dehydro-N-neuraminic acid
4GP	0.564	9.60	4.25	beta-D-aldopyranose	4c1	yes	N-(carboxycarbonyl)-beta-D-glucopyranosylamine
6GP	0.567	27.78	4.98	beta-D-aldopyranose	4c1	yes	N-[methoxy(oxo)acetyl]-beta-D-glucopyranosylamine
6MN	0.556	239.61	6.04	alpha-D-aldopyranose	4c1	yes	2-amino-2-deoxy-6-O-phosphono-alpha-D-mannopyranose
7JZ	0.554	315.73	5.29	beta-D-aldopyranose	4c1	yes	2-deoxy-2,2-difluoro-beta-D-lyxo-hexopyranose
8GP	0.566	19.44	4.88	beta-D-aldopyranose	4c1	yes	N-[(cyclopropylamino)(oxo)acetyl]-beta-D-glucopyranosylamine
A2G	0.541	261.50	9.77	alpha-D-aldopyranose	4c1	yes	2-acetamido-2-deoxy-alpha-D-galactopyranose
A6P	0.552	255.48	3.24	alpha-D-aldopyranose	4c1	yes	6-O-phosphono-alpha-D-allopyranose
ABE	0.552	305.94	2.11	alpha-D-aldopyranose	4c1	yes	alpha-D-Abequopyranose
ADA	0.546	270.30	4.39	alpha-D-aldopyranose	4c1	yes	alpha-D-galactopyranuronic acid
AGL	0.551	292.99	3.10	alpha-D-aldopyranose	4c1	yes	4-amino-4,6-dideoxy-alpha-D-glucopyranose
AMN	0.539	33.50	176.30	alpha-L-ketopyranose	1c4	yes	methyl 5-acetamido-9-amino-3,5,9-trideoxy-D-glycero-alpha-D-galacto-non-2-ulopyranosidonic acid
AMU	0.562	305.63	2.62	beta-D-aldopyranose	4c1	yes	N-acetyl-beta-muramic acid
AMV	0.575	13.82	3.13	beta-D-aldopyranose	4c1	yes	methyl 2-acetamido-3-O-[(1R)-1-carboxyethyl]-2-deoxy-beta-D-glucopyranoside
ANA	0.542	40.04	175.76	alpha-L-ketopyranose	1c4	yes	methyl 4-O-acetyl-5-acetamido-3,5-dideoxy-D-glycero-alpha-D-galacto-non-2-ulopyranosidonic acid
ARA	0.557	336.74	4.85	alpha-N-aldopyranose	4c1	yes	alpha-L-arabinopyranose
ARW	0.570	15.06	2.28	beta-N-aldopyranose	4c1	yes	methyl beta-D-arabinopyranoside

ASG	0.556	323.57	3.82	beta-D-aldopyranose	4c1	yes	2-acetamido-2-deoxy-4-O-sulfo-beta-D-galactopyranose
ASO	0.568	351.87	2.88	beta-D-aldopyranose	4c1	yes	1,5-anhydro-D-glucitol
B16	0.566	329.64	3.43	beta-D-aldopyranose	4c1	yes	1,6-di-O-phosphono-beta-D-glucopyranose
B7G	0.567	15.10	3.50	beta-D-aldopyranose	4c1	yes	heptyl beta-D-glucopyranoside
BDG	0.550	238.39	4.87	alpha-D-aldopyranose	4c1	yes	2,6-diamino-2,6-dideoxy-alpha-D-glucopyranose
BDP	0.554	327.33	4.34	beta-D-aldopyranose	4c1	yes	beta-D-glucopyranuronic acid
BEM	0.564	333.78	2.56	beta-D-aldopyranose	4c1	yes	beta-D-mannopyranuronic acid
BG6	0.554	315.30	4.13	beta-D-aldopyranose	4c1	yes	6-O-phosphono-beta-D-glucopyranose
BGC	0.554	316.40	3.92	beta-D-aldopyranose	4c1	yes	beta-D-glucopyranose
BGL	0.567	345.41	1.08	beta-D-aldopyranose	4c1	yes	2-O-octyl-beta-D-glucopyranose
BGN	0.565	305.91	2.69	beta-D-aldopyranose	4c1	yes	2-(butanoylamino)-2-deoxy-beta-D-glucopyranose
BGP	0.560	289.86	7.25	beta-D-aldopyranose	4c1	yes	6-O-phosphono-beta-D-galactopyranose
BGS	0.563	346.40	4.17	beta-D-aldopyranose	4c1	yes	(1S)-1,5-anhydro-1-(ethylsulfonyl)-D-glucitol
BHG	0.567	338.47	3.60	beta-D-aldopyranose	4c1	yes	hexyl beta-D-galactopyranoside
BM3	0.538	256.00	9.53	alpha-D-aldopyranose	4c1	yes	2-acetamido-2-deoxy-alpha-D-mannopyranose
BMA	0.557	349.91	3.55	beta-D-aldopyranose	4c1	yes	beta-D-mannopyranose
BNG	0.563	353.62	3.07	beta-D-aldopyranose	4c1	yes	nonyl beta-D-glucopyranoside
BOG	0.565	358.18	3.46	beta-D-aldopyranose	4c1	yes	octyl beta-D-glucopyranoside
C3X	0.567	3.31	2.43	beta-N-aldopyranose	4c1	yes	(2R)-oxiran-2-ylmethyl beta-D-xylopyranoside
C4X	0.568	13.85	2.58	beta-N-aldopyranose	4c1	yes	3,4-epoxybutyl-beta-D-xyloside
C5X	0.567	3.18	2.54	beta-N-aldopyranose	4c1	yes	3-[(2R)-oxiran-2-yl]propyl beta-D-xylopyranoside
CBF	0.533	253.47	6.92	alpha-D-aldopyranose	4c1	yes	(2R,3R,4S,5S,6R)-2,3,4,5-tetrahydroxy-6-(hydroxymethyl)oxane-2-carboxamide
CDG	0.566	351.87	3.15	beta-D-aldopyranose	4c1	yes	methyl 4,6-O-[(1R)-1-carboxyethylidene]-beta-D-galactopyranoside

CEG	0.565	288.24	6.01	beta-D-aldopyranose	4c1	yes	4,6-O-[(1S)-1-carboxyethylidene]-beta-D-glucopyranose
CNP	0.540	52.29	174.60	alpha-L-ketopyranose	1c4	yes	2-propenyl-N-acetyl-neuraminic acid
CR1	0.562	7.78	4.75	beta-D-aldopyranose	4c1	yes	N-(methoxycarbonyl)-beta-D-glucopyranosylamine
CR6	0.536	246.33	8.55	beta-D-aldopyranose	4c1	yes	1-deoxy-1-acetylamino-beta-D-gluco-2-heptulopyranosonamide
CRA	0.543	204.12	6.97	beta-D-aldopyranose	4c1	yes	1-deoxy-1-methoxycarbamido-beta-D-gluco-2-heptulopyranosonamide
D6G	0.543	263.84	4.07	alpha-D-aldopyranose	4c1	yes	2-deoxy-6-O-phosphono-alpha-D-glucopyranose
DAG	0.558	319.16	5.82	beta-D-aldopyranose	4c1	yes	4-amino-4,6-dideoxy-beta-D-glucopyranose
DDA	0.552	316.07	5.27	beta-D-aldopyranose	4c1	yes	beta-D-Olivopyranose
DDL	0.555	306.71	5.79	beta-D-aldopyranose	4c1	yes	2,6-dideoxy-beta-D-galactopyranose
DEG	0.548	254.68	2.66	alpha-D-aldopyranose	4c1	yes	butyl alpha-D-mannopyranoside
DK4	0.547	350.24	5.15	beta-D-aldopyranose	4c1	yes	1-(3-deoxy-3-fluoro-beta-D-glucopyranosyl)-5-fluoropyrimidine-2,4(1H,3H)-dione
DK5	0.535	335.00	6.19	beta-D-aldopyranose	4c1	yes	1-(2,3-dideoxy-3-fluoro-beta-D-arabino-hexopyranosyl)-4-[(phenylcarbonyl)amino]pyrimidin-2(1H)-one
DKX	0.547	354.37	5.06	beta-D-aldopyranose	4c1	yes	1-(3-deoxy-3-fluoro-beta-D-glucopyranosyl)pyrimidine-2,4(1H,3H)-dione
DKY	0.551	3.52	5.88	beta-D-aldopyranose	4c1	yes	1-(3-deoxy-3-fluoro-beta-D-glucopyranosyl)-4-[(phenylcarbonyl)amino]pyrimidin-2(1H)-one
DKZ	0.546	356.14	5.14	beta-D-aldopyranose	4c1	yes	4-amino-1-(3-deoxy-3-fluoro-beta-D-glucopyranosyl)pyrimidin-2(1H)-one
DL6	0.564	17.07	4.57	beta-D-aldopyranose	4c1	yes	N-(azidoacetyl)-beta-D-glucopyranosylamine
DLF	0.544	90.52	173.63	alpha-L-aldopyranose	1c4	yes	2-deoxy-alpha-L-fucopyranose
DO8	0.544	243.34	6.62	alpha-D-ketopyranose	4c1	yes	3-deoxy-8-O-phosphono-alpha-D-manno-oct-2-ulopyranosonic acid
DRI	0.547	326.88	3.85	beta-D-aldopyranose	4c1	yes	2,6-dideoxy-4-O-methyl-beta-D-glucopyranose
DSR	0.552	318.97	4.12	beta-D-aldopyranose	4c1	yes	2,6-dideoxy-4-thio-beta-D-allopyranose
DVC	0.520	117.97	172.73	alpha-L-aldopyranose	1c4	yes	(2R,4S,6S)-4-azanyl-4,6-dimethyl-oxane-2,5,5-triol
EAG	0.575	350.87	3.58	beta-D-aldopyranose	4c1	yes	2-aminoethyl 2-acetamido-2-deoxy-beta-D-glucopyranoside
EBG	0.553	233.65	2.46	alpha-D-aldopyranose	4c1	yes	2-[(2S)-oxiran-2-yl]ethyl alpha-D-glucopyranoside

EMP	0.548	350.26	4.58	alpha-N-aldopyranose	4c1	yes	2,4-dideoxy-4-(ethylamino)-3-O-methyl-alpha-L-threo-pentopyranose
EPG	0.555	250.89	1.76	alpha-D-aldopyranose	4c1	yes	(2R)-oxiran-2-ylmethyl alpha-D-glucopyranoside
EQP	0.560	184.81	175.36	alpha-L-ketopyranose	1c4	yes	(1R)-4-acetamido-1,5-anhydro-2,4-dideoxy-1-phosphono-D-glycero-D-galacto-octitol
F1P	0.547	297.95	177.05	beta-N-ketopyranose	1c4	yes	1-O-phosphono-beta-D-fructopyranose
FCA	0.554	270.33	5.45	alpha-D-aldopyranose	4c1	yes	alpha-D-fucopyranose
FCB	0.561	300.51	6.18	beta-D-aldopyranose	4c1	yes	beta-D-fucopyranose
FUC	0.553	89.18	175.16	alpha-L-aldopyranose	1c4	yes	alpha-L-fucopyranose
FUL	0.560	122.71	174.09	beta-L-aldopyranose	1c4	yes	beta-L-fucopyranose
G0S	0.566	352.88	4.84	beta-D-aldopyranose	4c1	yes	3-(beta-D-galactopyranosylthio)propanoic acid
G16	0.559	261.53	3.94	alpha-D-aldopyranose	4c1	yes	1,6-di-O-phosphono-alpha-D-glucopyranose
G1P	0.555	241.59	2.06	alpha-D-aldopyranose	4c1	yes	1-O-phosphono-alpha-D-glucopyranose
G2F	0.555	230.43	4.48	alpha-D-aldopyranose	4c1	yes	2-deoxy-2-fluoro-alpha-D-glucopyranose
G3F	0.552	5.55	4.25	beta-D-aldopyranose	4c1	yes	3-deoxy-3-fluoro-beta-D-glucopyranose
G4D	0.537	274.87	5.93	alpha-D-aldopyranose	4c1	yes	4-deoxy-alpha-D-glucopyranose
G4S	0.554	318.99	4.37	beta-D-aldopyranose	4c1	yes	4-O-sulfo-beta-D-galactopyranose
G6P	0.552	263.79	4.57	alpha-D-aldopyranose	4c1	yes	6-O-phosphono-alpha-D-glucopyranose
G6S	0.558	311.67	5.22	beta-D-aldopyranose	4c1	yes	6-O-sulfo-beta-D-galactopyranose
G7P	0.558	304.76	4.80	beta-D-aldopyranose	4c1	yes	6,7-dideoxy-7-phosphono-beta-D-gluco-heptopyranose
GAA	0.561	280.37	3.92	alpha-D-aldopyranose	4c1	yes	3-nitrophenyl alpha-D-galactopyranoside
GAF	0.553	244.64	4.46	alpha-D-aldopyranose	4c1	yes	2-deoxy-2-fluoro-alpha-D-galactopyranose
GAL	0.559	292.83	5.65	beta-D-aldopyranose	4c1	yes	beta-D-galactopyranose
GAT	0.559	167.72	1.14	alpha-D-aldopyranose	4c1	yes	4-aminophenyl alpha-D-galactopyranoside
GC4	0.544	344.07	4.54	beta-D-aldopyranose	4c1	yes	4-deoxy-beta-D-glucopyranuronic acid

GCN	0.535	278.16	4.66	alpha-D-aldopyranose	4c1	yes	2-amino-2,3-dideoxy-alpha-D-glucoyranose
GCS	0.565	289.39	3.79	beta-D-aldopyranose	4c1	yes	2-amino-2-deoxy-beta-D-glucopyranose
GCU	0.545	250.13	2.20	alpha-D-aldopyranose	4c1	yes	alpha-D-glucopyranuronic acid
GCV	0.534	271.22	5.98	alpha-D-aldopyranose	4c1	yes	4-O-methyl-alpha-D-glucopyranuronic acid
GCW	0.555	322.41	4.17	beta-D-aldopyranose	4c1	yes	4-O-methyl-beta-D-glucopyranuronic acid
GDA	0.559	330.06	5.09	beta-D-aldopyranose	4c1	yes	4-amino-4-deoxy-beta-D-glucopyranose
GFP	0.554	236.24	4.46	alpha-D-aldopyranose	4c1	yes	2-deoxy-2-fluoro-1-O-phosphono-alpha-D-glucopyranose
GL0	0.554	321.70	4.75	beta-D-aldopyranose	4c1	yes	beta-D-gulopyranose
GL1	0.556	260.38	2.41	alpha-D-aldopyranose	4c1	yes	1-O-phosphono-alpha-D-galactopyranose
GL2	0.532	194.81	5.60	beta-D-aldopyranose	4c1	yes	(5S,7R,8S,9S,10R)-3-amino-8,9,10-trihydroxy-7-(hydroxymethyl)-6-oxa-1,3-diazaspiro[4.5]decane-2,4-dione
GLA	0.551	263.76	4.39	alpha-D-aldopyranose	4c1	yes	alpha-D-galactopyranose
GLC	0.548	245.03	1.71	alpha-D-aldopyranose	4c1	yes	alpha-D-glucopyranose
GLD	0.546	279.14	3.04	alpha-D-aldopyranose	4c1	yes	4,6-dideoxy-alpha-D-xylo-hexopyranose
GLF	0.550	264.44	2.89	alpha-D-aldopyranose	4c1	yes	alpha-D-glucopyranosyl fluoride
GLG	0.544	262.48	8.47	alpha-D-aldopyranose	4c1	yes	Alpha-D-glucopyranosyl-2-carboxylic acid amide
GLP	0.556	244.30	6.21	alpha-D-aldopyranose	4c1	yes	2-amino-2-deoxy-6-O-phosphono-alpha-D-glucopyranose
GLS	0.534	211.43	5.10	beta-D-aldopyranose	4c1	yes	Beta-D-glucopyranose spirohydantoin
GMB	0.570	30.82	2.69	beta-D-aldopyranose	4c1	yes	1,7-di-O-phosphono-L-glycero-beta-D-manno-heptopyranose
GMH	0.545	278.34	3.06	alpha-D-aldopyranose	4c1	yes	L-glycero-alpha-D-manno-heptopyranose
GP4	0.550	238.87	4.40	alpha-D-aldopyranose	4c1	yes	2-amino-2-deoxy-4-O-phosphono-alpha-D-glucopyranose
GS1	0.563	352.72	4.33	beta-D-aldopyranose	4c1	yes	1-thio-beta-D-glucopyranose
GTM	0.554	2.72	4.09	beta-D-aldopyranose	4c1	yes	methyl 4-thio-beta-D-glucopyranoside
GTR	0.557	309.98	5.98	beta-D-aldopyranose	4c1	yes	beta-D-galactopyranuronic acid

GU0	0.568	305.59	1.59	beta-D-aldopyranose	4c1	yes	2,3,6-tri-O-sulfonato-beta-D-glucopyranose
GU3	0.562	251.91	1.48	alpha-D-aldopyranose	4c1	yes	methyl 3-O-methyl-2,6-di-O-sulfo-alpha-D-glucopyranoside
GU4	0.577	219.06	6.21	alpha-D-aldopyranose	4c1	yes	2,3,4,6-tetra-O-sulfonato-alpha-D-glucopyranose
GU8	0.574	61.68	3.43	beta-D-aldopyranose	4c1	yes	2,3,6-tri-O-methyl-beta-D-glucopyranose
GU9	0.503	262.07	167.03	alpha-D-aldopyranose	1c4	yes	2,3,6-tri-O-methyl-alpha-D-glucopyranose
GUP	0.537	119.59	175.27	alpha-L-aldopyranose	1c4	yes	alpha-L-gulopyranose
GXL	0.552	85.34	175.36	alpha-L-aldopyranose	1c4	yes	Alpha-L-galactopyranose
H1M	0.555	225.05	2.59	alpha-D-aldopyranose	4c1	yes	methyl 2-deoxy-2-(2-hydroxyethyl)-alpha-D-mannopyranoside
H2P	0.555	151.08	1.46	alpha-D-aldopyranose	4c1	yes	1-deoxy-2-O-phosphono-alpha-D-gluco-hept-2-ulopyranose
IDG	0.564	142.69	177.18	beta-L-aldopyranose	1c4	yes	2,6-diamino-2,6-dideoxy-beta-L-idopyranose
IDR	0.540	102.30	175.54	alpha-L-aldopyranose	1c4	yes	alpha-L-idopyranuronic acid
IMK	0.560	319.96	3.48	beta-D-aldopyranose	4c1	yes	2-(beta-D-glucopyranosyl)-5-methyl-1-benzimidazole
IPT	0.567	346.35	4.37	beta-D-aldopyranose	4c1	yes	1-methylethyl 1-thio-beta-D-galactopyranoside
JHM	0.536	272.67	9.83	alpha-D-aldopyranose	4c1	yes	2-deoxy-6-O-sulfo-alpha-D-glucopyranose
JZR	0.565	339.54	2.98	beta-D-aldopyranose	4c1	yes	hexyl beta-D-glucopyranoside
KDA	0.552	267.18	3.87	alpha-D-ketopyranose	4c1	yes	prop-2-en-1-yl 3-deoxy-alpha-D-manno-oct-2-ulopyranosidonic acid
KDB	0.445	315.41	48.84	beta-D-ketopyranose	Oh5	yes	3,4,5-trideoxy-alpha-D-erythro-oct-3-en-2-ulopyranosonic acid
KDO	0.543	238.68	5.25	beta-D-ketopyranose	4c1	yes	3-deoxy-alpha-D-manno-oct-2-ulopyranosonic acid
KDR	0.554	268.11	3.59	beta-D-ketopyranose	4c1	yes	prop-2-en-1-yl 3-deoxy-alpha-D-manno-octos-2-ulopyranoside
KME	0.554	284.38	2.61	alpha-D-ketopyranose	4c1	yes	(1E)-prop-1-en-1-yl 3-deoxy-7-O-methyl-alpha-D-manno-oct-2-ulopyranosidonic acid
KOT	0.571	36.10	1.86	beta-D-aldopyranose	4c1	yes	1-beta-D-glucopyranosyl-4-phenyl-1H-1,2,3-triazole
L6S	0.550	88.07	177.81	alpha-L-aldopyranose	1c4	yes	6-O-sulfo-alpha-L-galactopyranose
LGU	0.536	93.17	174.07	alpha-L-aldopyranose	1c4	yes	alpha-L-gulopyranuronic acid

LXB	0.561	312.87	4.14	beta-D-aldopyranose	4c1	yes	2-acetamido-2-deoxy-beta-D-gulopyranose
LXC	0.561	151.16	176.06	beta-N-aldopyranose	1c4	yes	Beta-L-xylopyranose
LXZ	0.541	270.80	6.85	alpha-D-aldopyranose	4c1	yes	2-acetamido-2-deoxy-alpha-D-idopyranose
LZ0	0.559	75.44	175.73	alpha-L-aldopyranose	1c4	yes	[1-(2-oxoethyl)-1H-1,2,3-triazol-5-yl]methyl 6-deoxy-alpha-L-galactopyranoside
M07	0.537	201.48	3.79	alpha-D-aldopyranose	4c1	yes	(5R,7R,8S,9S,10R)-7-(hydroxymethyl)-3-(4-methoxyphenyl)-1,6-dioxa-2-azaspiro[4.5]dec-2-ene-8,9,10-triol
M08	0.538	201.20	3.79	alpha-D-aldopyranose	4c1	yes	(5R,7R,8S,9S,10R)-7-(hydroxymethyl)-3-phenyl-1,6-dioxa-2-azaspiro[4.5]dec-2-ene-8,9,10-triol
M09	0.539	227.02	3.64	alpha-D-aldopyranose	4c1	yes	(3S,5R,7R,8S,9S,10R)-7-(hydroxymethyl)-3-(4-nitrophenyl)-1,6-dioxa-2-azaspiro[4.5]decane-8,9,10-triol
M1P	0.549	263.28	1.69	alpha-D-aldopyranose	4c1	yes	1-O-phosphono-alpha-D-mannopyranose
M6D	0.562	355.95	1.99	beta-D-aldopyranose	4c1	yes	6-O-phosphono-beta-D-mannopyranose
M7P	0.553	269.62	6.46	beta-D-aldopyranose	4c1	yes	7-O-phosphono-D-glycero-alpha-D-manno-heptopyranose
M8C	0.549	266.30	5.30	alpha-D-aldopyranose	4c1	yes	methyl alpha-D-galactopyranuronate
MA1	0.550	257.24	1.68	alpha-D-aldopyranose	4c1	yes	1,4-dithio-alpha-D-glucopyranose
MA2	0.549	272.99	7.66	alpha-D-aldopyranose	4c1	yes	4-S-methyl-4-thio-alpha-D-glucopyranose
MA3	0.550	272.33	0.99	alpha-D-aldopyranose	4c1	yes	methyl 4-thio-alpha-D-glucopyranoside
MAG	0.573	346.83	3.41	beta-D-aldopyranose	4c1	yes	methyl 2-acetamido-2-deoxy-beta-D-glucopyranoside
MAN	0.538	260.65	5.99	alpha-D-aldopyranose	4c1	yes	alpha-D-mannopyranose
MAT	0.524	112.14	172.74	alpha-N-aldopyranose	1c4	yes	2,4-dideoxy-3-O-methyl-4-(propan-2-ylamino)-alpha-L-threo-pentopyranose
MAV	0.545	237.87	2.46	alpha-D-aldopyranose	4c1	yes	alpha-D-mannopyranuronic acid
MBF	0.561	297.37	2.58	beta-D-aldopyranose	4c1	yes	2-deoxy-2-fluoro-beta-D-mannopyranose
MBG	0.567	334.98	3.74	beta-D-aldopyranose	4c1	yes	methyl beta-D-galactopyranoside
MDA	0.551	335.67	4.63	beta-D-aldopyranose	4c1	yes	2,6-dideoxy-3-C-methyl-beta-D-ribo-hexopyranose
MDP	0.563	322.01	3.58	beta-D-aldopyranose	4c1	yes	N-carboxyl-N-methyl-beta-muramic acid
MFB	0.570	120.95	176.50	beta-L-aldopyranose	1c4	yes	methyl beta-L-fucopyranoside

MGC	0.565	279.53	1.57	alpha-D-aldopyranose	4c1	yes	methyl 2-acetamido-2-deoxy-alpha-D-galactopyranoside
MGL	0.565	348.90	3.08	beta-D-aldopyranose	4c1	yes	methyl beta-D-glucopyranoside
MGS	0.551	292.07	3.16	alpha-D-aldopyranose	4c1	yes	methyl 4,6-dideoxy-4-{[(2R)-2,4-dihydroxybutanoyl]amino}-2-O-methyl-alpha-D-mannopyranoside
MMA	0.557	259.64	2.21	alpha-D-aldopyranose	4c1	yes	methyl alpha-D-mannopyranoside
MNA	0.541	33.61	176.01	alpha-L-ketopyranose	1c4	yes	2-O-methyl-5-N-acetyl-alpha-D-neuraminic acid
MQT	0.578	322.92	2.79	beta-D-aldopyranose	4c1	yes	methyl 2-O-acetyl-3-O-(4-methylbenzoyl)-beta-D-talopyranoside
MRP	0.551	92.30	177.66	alpha-L-aldopyranose	1c4	yes	3-O-methyl-alpha-L-rhamnopyranose
MUR	0.562	293.29	4.65	beta-D-aldopyranose	4c1	yes	beta-muramic acid
MXZ	0.578	302.54	176.41	alpha-L-aldopyranose	1c4	yes	2-O-methyl-alpha-L-fucopyranose
NAA	0.565	299.76	2.55	beta-D-aldopyranose	4c1	yes	2-acetamido-2-deoxy-beta-D-allopyranose
NAG	0.564	300.23	2.93	beta-D-aldopyranose	4c1	yes	2-acetamido-2-deoxy-beta-D-glucopyranose
NBG	0.564	9.27	4.48	beta-D-aldopyranose	4c1	yes	N-acetyl-beta-D-glucopyranosylamine
NDG	0.550	244.22	5.24	alpha-D-aldopyranose	4c1	yes	2-acetamido-2-deoxy-alpha-D-glucopyranose
NG1	0.550	263.29	4.96	alpha-D-aldopyranose	4c1	yes	2-acetamido-2-deoxy-1-O-phosphono-alpha-D-galactopyranose
NG6	0.564	329.01	2.94	beta-D-aldopyranose	4c1	yes	2-acetamido-2-deoxy-6-O-sulfo-beta-D-galactopyranose
NGA	0.562	307.41	4.41	beta-D-aldopyranose	4c1	yes	2-acetamido-2-deoxy-beta-D-galactopyranose
NGK	0.546	263.09	6.19	alpha-D-aldopyranose	4c1	yes	2-acetamido-2-deoxy-4-O-sulfo-alpha-D-galactopyranose
NGZ	0.551	55.73	175.71	alpha-L-aldopyranose	1c4	yes	2-acetamido-2-deoxy-alpha-L-glucopyranose
NNG	0.557	229.00	4.63	alpha-D-aldopyranose	4c1	yes	2-deoxy-2-{[(S)-hydroxy(methyl)phosphoryl]amino}-6-O-phosphono-alpha-D-glucopyranose
NOK	0.569	71.73	3.73	beta-D-aldopyranose	4c1	yes	2-acetamido-1,2-dideoxynojirmycin
NTF	0.564	8.02	4.30	beta-D-aldopyranose	4c1	yes	N-(trifluoroacetyl)-beta-D-glucopyranosylamine
NXD	0.545	49.28	175.95	beta-L-ketopyranose	1c4	yes	methyl 5-acetamido-9-{[amino(oxo)acetyl]amino}-3,5,9-trideoxy-D-glycero-alpha-D-galacto-non-2-ulopyranosidonic acid
OAK	0.567	31.24	5.66	beta-D-aldopyranose	4c1	yes	N-(phenylcarbonyl)-beta-D-glucopyranosylamine

OPM	0.549	257.55	2.17	alpha-D-aldopyranose	4c1	yes	pentyl alpha-D-mannopyranoside
OTG	0.539	252.54	7.21	alpha-D-aldopyranose	4c1	yes	2-deoxy-2-{[(2-methylphenyl)carbonyl]amino}-alpha-D-glucopyranose
OX2	0.566	332.14	2.73	beta-D-aldopyranose	4c1	yes	(1R)-1,5-anhydro-1-(5-methyl-1,3,4-oxadiazol-2-yl)-D-glucitol
PA1	0.554	234.78	4.63	alpha-D-aldopyranose	4c1	yes	2-amino-2-deoxy-alpha-D-glucopyranose
PDX	0.555	196.44	1.26	alpha-D-aldopyranose	4c1	yes	2,3-di-O-sulfo-alpha-D-glucopyranose
PH5	0.541	32.39	175.80	alpha-L-ketopyranose	1c4	yes	benzyl 3,5-dideoxy-5-(propanoylamino)-D-glycero-alpha-D-galacto-non-2-ulopyranosidonic acid
PNA	0.558	251.39	3.29	alpha-D-aldopyranose	4c1	yes	4-nitrophenyl alpha-D-mannopyranoside
PNG	0.558	247.45	1.78	alpha-D-aldopyranose	4c1	yes	4-nitrophenyl alpha-D-glucopyranoside
PNJ	0.566	319.97	4.34	beta-D-aldopyranose	4c1	yes	4-nitrophenyl 2-amino-2-deoxy-beta-D-glucopyranoside
PNW	0.573	357.34	3.30	beta-D-aldopyranose	4c1	yes	4-nitrophenyl beta-D-glucopyranoside
PSG	0.560	3.32	3.74	beta-D-aldopyranose	4c1	yes	4-nitrophenyl 1-thio-beta-D-glucopyranoside
RAM	0.551	93.67	175.46	alpha-L-aldopyranose	1c4	yes	alpha-L-rhamnopyranose
RAO	0.560	74.22	177.17	alpha-L-aldopyranose	1c4	yes	methyl 6-deoxy-alpha-L-rhamnopyranoside
RER	0.508	92.81	172.03	alpha-L-aldopyranose	1c4	yes	vancosamine
RGG	0.567	343.74	3.59	beta-D-aldopyranose	4c1	yes	(2R)-2,3-dihydroxypropyl beta-D-galactopyranoside
RIP	0.564	333.38	2.88	beta-N-aldopyranose	4c1	yes	beta-D-ribopyranose
RM4	0.570	134.59	176.74	beta-L-aldopyranose	1c4	yes	Beta-L-rhamnopyranose
RUG	0.573	46.59	2.13	beta-D-aldopyranose	4c1	yes	1-beta-D-glucopyranosyl-4-(hydroxymethyl)-1H-1,2,3-triazole
S06	0.539	227.03	3.51	alpha-D-aldopyranose	4c1	yes	(3S,5R,7R,8S,9S,10R)-7-(hydroxymethyl)-3-(2-naphthyl)-1,6-dioxa-2-azaspiro[4.5]decane-8,9,10-triol
S13	0.540	227.54	3.66	alpha-D-aldopyranose	4c1	yes	(3S,5R,7R,8S,9S,10R)-7-(hydroxymethyl)-3-(4-methylphenyl)-1,6-dioxa-2-azaspiro[4.5]decane-8,9,10-triol
SFU	0.570	176.94	177.84	alpha-L-aldopyranose	1c4	yes	methyl 1-seleno-alpha-L-fucopyranoside
SGA	0.564	295.07	3.87	beta-D-aldopyranose	4c1	yes	3-O-sulfo-beta-D-galactopyranose
SGC	0.553	346.52	4.24	beta-D-aldopyranose	4c1	yes	4-thio-beta-D-glucopyranose

SGN	0.543	255.36	9.21	alpha-D-aldopyranose	4c1	yes	2-deoxy-6-O-sulfo-2-(sulfoamino)-alpha-D-glucopyranose
SHG	0.562	286.68	3.36	beta-D-aldopyranose	4c1	yes	2-deoxy-2-fluoro-beta-D-glucopyranose
SIA	0.539	61.92	175.13	alpha-L-ketopyranose	1c4	yes	N-acetyl-alpha-neuraminic acid
SID	0.542	41.16	176.14	alpha-L-ketopyranose	1c4	yes	methyl 9-S-acetyl-5-acetamido-3,5-dideoxy-9-thio-D-glycero-alpha-D-galacto-non-2-ulopyranosidonic acid
SLB	0.539	69.21	175.93	beta-L-ketopyranose	1c4	yes	N-acetyl-beta-neuraminic acid
SLM	0.543	65.58	175.84	beta-L-ketopyranose	1c4	yes	(2S,4S,5R,6R)-5-acetamido-2,4-dihydroxy-6-[(1R,2R)-1,2,3-trihydroxypropyl]oxane-2-carboxamide
SN5	0.563	303.83	3.63	beta-D-aldopyranose	4c1	yes	2-deoxy-2-(ethanethioylamino)-beta-D-glucopyranose
SOE	0.551	321.70	176.81	alpha-N-ketopyranose	1c4	yes	alpha-L-sorbopyranose
SOG	0.564	4.35	4.65	beta-D-aldopyranose	4c1	yes	octyl 1-thio-beta-D-glucopyranoside
SSG	0.559	5.99	4.84	beta-D-aldopyranose	4c1	yes	1,4-dithio-beta-D-glucopyranose
STZ	0.563	298.49	3.44	beta-D-aldopyranose	4c1	yes	streptozotocin
SUS	0.550	194.66	2.29	alpha-D-aldopyranose	4c1	yes	2-deoxy-3,6-di-O-sulfo-2-(sulfoamino)-alpha-D-glucopyranose
TGA	0.567	331.97	3.56	beta-D-aldopyranose	4c1	yes	2-sulfanylethyl beta-D-galactopyranoside
TMR	0.555	313.66	3.21	beta-D-aldopyranose	4c1	yes	2,6-dideoxy-4-S-methyl-4-thio-beta-D-ribo-hexopyranose
TOA	0.534	286.20	4.28	alpha-D-aldopyranose	4c1	yes	3-ammonio-3-deoxy-alpha-D-glucopyranose
TOC	0.538	299.00	3.14	alpha-D-aldopyranose	4c1	yes	2,6-diammonio-2,3,6-trideoxy-alpha-D-glucopyranose
TYV	0.550	304.50	1.43	alpha-D-aldopyranose	4c1	yes	alpha-D-Tyvelopyranose
X1P	0.555	228.40	1.07	alpha-N-aldopyranose	4c1	yes	1-O-phosphono-alpha-D-xylopyranose
XYP	0.560	329.52	4.07	beta-N-aldopyranose	4c1	yes	Beta-D-xylopyranose
XYS	0.554	259.31	1.86	alpha-N-aldopyranose	4c1	yes	alpha-D-xylopyranose
YX0	0.558	83.60	177.05	alpha-L-aldopyranose	1c4	yes	[(3E)-3-(1-hydroxyethylidene)-2,3-dihydroisoxazol-5-yl]methyl alpha-L-fucopyranoside
YX1	0.569	291.93	5.27	beta-D-aldopyranose	4c1	yes	2-deoxy-2-{[(2-hydroxy-1-methylhydrazino)carbonyl]amino}-beta-D-glucopyranose
